# Knowing Where You Are Going: Co-Producing and Standardising Information About Child and Adolescent Mental Health Services Inpatient Units

**DOI:** 10.1192/bjo.2024.424

**Published:** 2024-08-01

**Authors:** Josephine Holland, James Roe, Kapil Sayal

**Affiliations:** University of Nottingham, Nottingham, United Kingdom

## Abstract

**Aims:**

At-distance and out-of-region admissions form a significant proportion of inpatient admissions in CAMHS. The recent national “Far Away from Home” study which investigated the impacts of these admissions for young people, parents/carers and services identified an inconsistent and/or lack of easily accessible information about inpatient units. Parents and young people reported that when there was a lack of easily accessible information about the unit they would be admitted to, this increased their distress and negative views about the admission before they had even arrived. In contrast, those who found useful and positive information felt more reassured about the admission, even if it was far away. Our aim was to create an expert-by-experience designed standardised template of the minimum information that all inpatient units would be required to make publicly available.

**Methods:**

We carried out regular expert consultation meetings with young people and parents/carers with lived experience to co-design a standardised template of information that units would provide for young people and their families on their websites and in printed form.

**Results:**

In early meetings the information currently presented by inpatient units was reviewed and discussed. Young people and parents/carers highlighted what they found helpful and unhelpful as well as what was missing. The young people and parents/carers discussed the layout, aesthetics, and functionality that they would like to see on unit websites. They also discussed the content which would be helpful for young people and their parents individually as well as what both groups would want to know. This included realistic and practical information about the unit itself, visiting, local amenities and available funding support.

**Conclusion:**

In collaboration with young people and parents/carers we have created an expert-by-experience designed standardised template of information that all inpatient units will be asked to provide on their website. Better information provision prior to admission will reduce anxiety and uncertainty for young people and their families. We anticipate this project will also contribute towards improved staff/patient/carer relationships because of clearer expectations and understanding.

